# Reduced nucleus accumbens functional connectivity in reward network and default mode network in patients with recurrent major depressive disorder

**DOI:** 10.1038/s41398-022-01995-x

**Published:** 2022-06-06

**Authors:** Yu-Dan Ding, Xiao Chen, Zuo-Bing Chen, Le Li, Xue-Ying Li, Francisco Xavier Castellanos, Tong-Jian Bai, Qi-Jing Bo, Jun Cao, Zhi-Kai Chang, Guan-Mao Chen, Ning-Xuan Chen, Wei Chen, Chang Cheng, Yu-Qi Cheng, Xi-Long Cui, Jia Duan, Yi-Ru Fang, Qi-Yong Gong, Zheng-Hua Hou, Lan Hu, Li Kuang, Feng Li, Hui-Xian Li, Kai-Ming Li, Tao Li, Yan-Song Liu, Zhe-Ning Liu, Yi-Cheng Long, Bin Lu, Qing-Hua Luo, Hua-Qing Meng, Dai-Hui Peng, Hai-Tang Qiu, Jiang Qiu, Yue-Di Shen, Yu-Shu Shi, Tian-Mei Si, Yan-Qing Tang, Chuan-Yue Wang, Fei Wang, Kai Wang, Li Wang, Xiang Wang, Ying Wang, Yu-Wei Wang, Xiao-Ping Wu, Xin-Ran Wu, Chun-Ming Xie, Guang-Rong Xie, Hai-Yan Xie, Peng Xie, Xiu-Feng Xu, Hong Yang, Jian Yang, Jia-Shu Yao, Shu-Qiao Yao, Ying-Ying Yin, Yong-Gui Yuan, Yu-Feng Zang, Ai-Xia Zhang, Hong Zhang, Ke-Rang Zhang, Lei Zhang, Zhi-Jun Zhang, Jing-Ping Zhao, Ru-Bai Zhou, Yi-Ting Zhou, Jun-Juan Zhu, Zhi-Chen Zhu, Chao-Jie Zou, Xi-Nian Zuo, Chao-Gan Yan, Wen-Bin Guo

**Affiliations:** 1 Department of Psychiatry, and National Clinical Research Center for Mental Disorders, The Second Xiangya Hospital of Central South University, Changsha, China; 2 CAS Key Laboratory of Behavioral Science, Institute of Psychology, Beijing, China; 3 Department of Psychology, University of Chinese Academy of Sciences, Beijing, China; 4 International Big-Data Center for Depression Research, Chinese Academy of Sciences, Beijing, China; 5 Department of Rehabilitation Medicine, The First Affiliated Hospital, College of Medicine, Zhejiang University, Hangzhou, Zhejiang China; 6 Center for Cognitive Science of Language, Beijing Language and Culture University, Beijing, China; 7 Sino-Danish College, University of Chinese Academy of Sciences, Beijing, China; 8 Sino-Danish Center for Education and Research, Beijing, China; 9 Department of Child and Adolescent Psychiatry, NYU Grossman School of Medicine, New York, 31 NY USA; 10 Nathan Kline Institute for Psychiatric Research, Orangeburg, NY USA; 11 Anhui Medical University, Hefei, Anhui China; 12 Beijing Anding Hospital, Capital Medical University, Beijing, China; 13 Department of Psychiatry, The First Affiliated Hospital of Chongqing Medical University, Chongqing, China; 14 The First Affiliated Hospital of Jinan University, Guangzhou, Guangdong China; 15 Sir Run Run Shaw Hospital, Zhejiang University School of Medicine, Hangzhou, Zhejiang China; 16 Department of Radiology, The First Affiliated Hospital, College of Medicine, Zhejiang University, Hangzhou, Zhejiang China; 17 Department of Psychiatry, First Affiliated Hospital of Kunming Medical University, Kunming, Yunnan China; 18 Department of Psychiatry, First Affiliated Hospital, China Medical University, Shenyang, Liaoning China; 19 Department of Psychiatry, Shanghai Jiao Tong University School of Medicine, Shanghai, China; 20 Huanxi MR Research Center (HMRRC), Department of Radiology, West China Hospital of Sichuan University, Chengdu, Sichuan China; 21 Psychoradiology Research Unit of Chinese Academy of Medical Sciences, West China Hospital of Sichuan University, Chengdu, Sichuan China; 22 Department of Psychosomatics and Psychiatry, Zhongda Hospital, School of Medicine, Southeast University, Nanjing, Jiangsu China; 23 Mental Health Center, West China Hospital, Sichuan University, Chengdu, Sichuan China; 24 Department of Clinical Psychology, Suzhou Psychiatric Hospital, The Affiliated Guangji Hospital of Soochow University, Suzhou, Jiangsu China; 25 Faculty of Psychology, Southwest University, Chongqing, China; 26 Department of Diagnostics, Affiliated Hospital, Hangzhou Normal University Medical School, Hangzhou, Zhejiang China; 27 National Clinical Research Center for Mental Disorders (Peking University Sixth Hospital) & Key Laboratory of Mental Health, Ministry of Health (Peking University), Beijing, China; 28 Xi’an Central Hospital, Xi’an, Shaanxi China; 29 Department of Neurology, Affiliated ZhongDa Hospital of Southeast University, Nanjing, Jiangsu China; 30 Department of Psychiatry, The Fourth Affiliated Hospital, College of Medicine, Zhejiang University, Yiwu, Zhejiang China; 31 Institute of Neuroscience, Chongqing Medical University, Chongqing, China; 32 Chongqing Key Laboratory of Neurobiology, Chongqing, China; 33 Department of Neurology, The First Affiliated Hospital of Chongqing Medical University, Chongqing, China; 34 Center for Cognition and Brain Disorders, Institutes of Psychological Sciences, Hangzhou Normal University, Hangzhou, Zhejiang China; 35 Zhejiang Key Laboratory for Research in Assessment of Cognitive Impairments, Hangzhou, Zhejiang China; 36 The First Affiliated Hospital of Xi’an Jiaotong University, Xi’an, Shaanxi China; 37 First Hospital of Shanxi Medical University, Taiyuan, Shanxi China; 38 Laboratory of Psychological Health and Imaging, Shanghai Mental Health Center, Shanghai Jiao Tong University School of Medicine, Shanghai, China; 39 State Key Laboratory of Cognitive Neuroscience and Learning & IDG/McGovern Institute for Brain Research, Beijing Normal University, Beijing, China; 40 Magnetic Resonance Imaging Research Center, Institute of Psychology, Chinese Academy of Sciences, Beijing, China

**Keywords:** Depression, Diagnostic markers

## Abstract

The nucleus accumbens (NAc) is considered a hub of reward processing and a growing body of evidence has suggested its crucial role in the pathophysiology of major depressive disorder (MDD). However, inconsistent results have been reported by studies on reward network-focused resting-state functional MRI (rs-fMRI). In this study, we examined functional alterations of the NAc-based reward circuits in patients with MDD via meta- and mega-analysis. First, we performed a coordinated-based meta-analysis with a new SDM-PSI method for all up-to-date rs-fMRI studies that focused on the reward circuits of patients with MDD. Then, we tested the meta-analysis results in the REST-meta-MDD database which provided anonymous rs-fMRI data from 186 recurrent MDDs and 465 healthy controls. Decreased functional connectivity (FC) within the reward system in patients with recurrent MDD was the most robust finding in this study. We also found disrupted NAc FCs in the DMN in patients with recurrent MDD compared with healthy controls. Specifically, the combination of disrupted NAc FCs within the reward network could discriminate patients with recurrent MDD from healthy controls with an optimal accuracy of 74.7%. This study confirmed the critical role of decreased FC in the reward network in the neuropathology of MDD. Disrupted inter-network connectivity between the reward network and DMN may also have contributed to the neural mechanisms of MDD. These abnormalities have potential to serve as brain-based biomarkers for individual diagnosis to differentiate patients with recurrent MDD from healthy controls.

## Introduction

Major depressive disorder (MDD) with heterogeneous symptoms immensely impairs patients’ health and function, destroys patients’ occupations and life, and mounts a burden borne by the family and society [1]. This disorder is characterized by a high recurrent rate and a chronic deteriorating disease course. As high as 83.3% (at least 33.5%) of patients with MDD may experience recurrence within 6 months [2]. Each recurrence of MDD especially increases the risk of becoming chronic. Up to 70% of patients with MDD may suffer recurrences throughout their lifetime after the second major depressive episode and by 90% after the third or more recurrence [3]. Over the past decades, with the development of precision medicine, more and more studies have focused on searching for objective biomarkers and exploring subtle classifications to guide clinical decision-making and facilitate individualized therapy.

Magnetic resonance imaging (MRI) is one of the noninvasive neuroimaging techniques used extensively to elucidate the neural basis of psychiatric disorders. This technique may be a critical step toward differential diagnoses and treatment prediction. Previous behavioral and MRI studies have implicated altered reward-related processes (e.g., reward anticipation, acceptance, and motivation) in patients with MDD [4–6]. Brain areas such as the putamen, ventral tegmental area (VTA), ventral striatum, anterior cingulate cortex (ACC), and medial prefrontal cortex (mPFC) are the components of complicated reward network [7]. Notably, converging evidence has suggested that structural and functional disruptions of the nucleus accumbens (NAc, a part of the ventral striatum) might be a crucial culprit of reward-related abnormalities. The NAc is a key component of dopamine-rich mesocorticolimbic pathways. This region is extensively connected with other brain regions and receives complex signal inputs from various neurochemistry systems including glutamatergic, dopaminergic, serotoninergic, and histaminergic projections [8–10]. For instance, the NAc volumes were smaller in patients with life-time MDD than in healthy controls [11]. During a monetary incentive delay task, individuals with MDD displayed significantly weaker activations to gains in the left NAc and bilateral caudate regions [12]. Generally, decreased activation within the ventral or dorsal striatum, coupled with increased or decreased activation in other areas (such as the mPFC and subgeneual ACC) was the most consistent finding in adolescents with MDD in the reward-related task studies using functional MRI (fMRI) [13–15]. Similarly, aberrant functional connectivities (FCs) between the NAc and other regions within the reward network, including the ACC, orbital frontal cortex (OFC) and mPFC, have been reported in adult patients with MDD in the resting-state fMRI (rs-fMRI) studies. These disruptions have been associated with depressive severity, cognitive deficit, and treatment response to repetitive transcranial magnetic stimulation [16–19]. Intriguingly, some brain regions such as the mPFC were also involved in the default mode network (DMN), a network which plays a critical role in self-referential and internal goal-oriented processes [20, 21]. The balanced activity between the reward network and other networks, such as the cognitive control network and DMN, was considered important for self-regulation, emotional regulation and reward motivation, and decision-making [22–24]. Gong et al. [25] reported decreased FCs between the NAc and areas within the reward network (bilateral caudate, hippocampus, left pallidum, etc.) and key hubs of the DMN (inferior parietal lobe and dorsal mPFC) and cognitive network (dorsolateral PFC). Furthermore, decreased inter-network FC and diminished controlling of the cognitive network on the reward network were documented in this study, and the imbalance between these networks could predict the severity of anhedonia in patients with MDD.

However, inconsistent results have been reported in these reward network-focused rs-fMRI studies. For example, both increased and decreased FCs have been observed between the NAc and the precuneus and mPFC [16, 18, 25–27]. Diverse demographic and clinical profiles of the subjects, analytical methods, and limited statistical power may have contributed to the inconsistencies. Therefore, in the current study, we examined functional alterations in the NAc-based reward circuits in patients with MDD via meta-analysis and mega-analysis. First, we performed a neurofunctional meta-analysis of all up-to-date rs-fMRI studies that focused on the reward circuits of patients with MDD. Then, we tested the meta-analysis results in a large MDD rs-fMRI database, that is, REST-meta-MDD (rfmri.org/REST-meta-MDD). This project contains anonymous rs-fMRI data processed using a standard pipeline built-in Data Processing & Analysis for Brain Imaging/Data Processing Assistant for Resting-State fMRI (DPABI/DPARSF) of 1,300 patients with MDD and 1,128 healthy controls. With the use of these data, project initiators Yan et al. found reduced DMN FC only in patients with recurrent MDD [28]. In the present investigation, we predicted decreased NAc FC in the reward network and compromised relationships between the NAc-based reward network and other networks (e.g., DMN), and mega-analysis would replicate the meta-analysis findings. In addition, we tested whether the altered NAc FC would serve as a potential image feature to distinguish patients with MDD from healthy controls.

## Materials and methods

### Meta-analysis

#### Search strategy, selection criteria, and data extraction

The meta-analysis was prepared compliant with Preferred Reporting Items for Systematic Reviews and Meta-analyses guidelines. A literature search of the PubMed and Web of Science database was conducted in September 2021. The following keywords and their combinations were used in [Title/Abstract]: resting-state functional magnetic resonance imaging/rsfMRI, major depressive disorder/MDD/depression/unipolar depression/depressive disorders, and reward network/nucleus accumbens/NAc/ventral striatum/VS. The reference lists of previous review articles were also manually searched.

All original rs-fMRI studies that compared patients with MDD with healthy controls and investigated NAc/VS FC maps were included. Meeting abstracts, study protocols, reviews, and animal studies were excluded. Studies were also excluded if they used different thresholds in different brain areas (e.g., region of interest) or recruited subjects with other major psychiatric or neurological illness.

The following variables were extracted from included studies: sample size, age, sex, education level of patients and controls, depressive severity, type of analysis, statistical thresholds, effect sizes, and if available, the Montreal Neurological Institute space (MNI) or Talairach space coordinates of brain areas with significant group differences.

#### Statistical analysis

We used a new method, SDM-PSI, to conduct this coordinated-based meta-analysis. This method was developed by Radua et al., and they implemented the Permutation of Subject Images (PSI) algorithm to an existing Anisotropic Effect-Size Seed-based d Mapping (AES-SDM) method [29]. Based on the AES-SDM method [30, 31], a set of reported peak coordinates and *t*-values were converted into Hedge’s g effect size using standard formulas. This process imputes the slightly lower effect sizes of surrounding voxels than those of the peaks, and until the voxels are far enough from any peak it imputes the effect size as null. However, non-negligible biases would be yielded in the progressive estimation of the effect size of voxels farther from the peaks. To address this limitation, the SDM-PSI method uses AES-SDM Gaussian kernels to calculate the effect-size bounds, maximum-likelihood estimation to estimate parameters, and multiple imputation techniques for each voxel to cover the uncertainty linked to single imputation with a range of effect sizes a voxel may have [29]. Importantly, the MetaNSUE (nonstatistically significant unreported effects [NSUE]) method is adapted to avoid biases introduced by excluding studies with non-significant results and unknown statistics [32, 33].

Age and years of education were included as covariates in the analyses. Given the intra- and inter-study heterogeneities, random-effect models were used and *I*^*2*^ statistic was applied to quantify heterogeneity (*I*^*2*^ ≥ 50% indicates substantial heterogeneity). The threshold was set at *p* < 0.001 uncorrected and a cluster extent of ten voxels. According to previous studies [29], this particular set of threshold was a conservative recommendation for cases where the *t*-values were not reported by studies and simultaneously has a remarkable sensitivity and controls the empirical family-wise error rate below 5%. Funnel plots were used to detect whether results might have been biased by small studies. In addition, meta-regression by the mean MDD symptom severity and the percentage of male individuals in the patient group were complemented to the main analysis. All analyses were performed using the SDM-PSI version 6.21 (https://www.sdmproject.com/).

We also conducted a supplementary analysis for the studies only focused on the NAc rather than VS, given possible inconsistency introduced by a broader ROI selection.

### Mega-analysis

#### Subjects

From the data of 2428 subjects provided by the REST-meta-MDD consortium, we selected the data of 186 subjects with recurrent MDDs and 465 matched healthy controls from eight sites across China (the majority of patients included in the meta-analysis were recurrent MDDs. Thus, we focused on recurrent MDDs). The sample were selected as shown in Fig. S1. All individuals aged 18–65 years with complete information (i.e., age, sex, and educational level) were included. Subjects were excluded if they (1) had poor imaging quality or poor spatial normalization (checked by visual inspection), (2) were in remission stage (the score of the 17-item Hamilton depression rating scale (HAMD) ≤7), or (3) had excessive head motion (mean framewise displacement, FD >0.2 mm). Among the 803 remaining patients with MDD, 186 were patients with recurrent MDDs. Data on the duration of illness and 17-item HAMD scores were available for 165 and 143 patients with recurrent MDDs, respectively. Medication information was provided by 126 patients with recurrent MDD, among which 77 patients were taking antidepressants when receiving MRI scans.

#### Data acquisition, preprocessing, and FC analysis

MRI scans and data preprocessing were performed independently at each site. The scanning parameters of each site and preprocessing details are shown in the Supplementary Materials. As previously described in refs. [28, 34], all preprocessing steps were completed with the DPARSF software, and analysts from each site were able to use it skillfully.

After preprocessing, time series for the Harvard-Oxford Atlas were extracted. We selected bilateral NAc as seed regions and calculated FC (Fisher’s z-transformed Pearson’s correlation coefficients) between the seeds and other brain areas.

#### Statistical analysis

Bilateral NAc FC maps of patients with recurrent MDD and controls were compared by linear mixed models (LMM) to control for potential study site-related effects, in which the group, age, sex, years of education, and mean FD were included as independent variables: y~1 + group + age + sex + years of education + FD + (1 | site) + (group | site) [28]. Then the yielded *t* and *df* values were used to calculate effect size Cohen’s *d* = (t(*n*1 + *n*2)) ⁄ (√df)√*n*1*n*2) [35]. To further test the potential effect of the depressive severity on the brain regions with significant group differences, we replaced “group” in the LMM model with HAMD total score or score of core depression subtype (item 1 and 7) [36]. The Pearson’s correlation coefficient r = t / √(n – 2 + t^2^) [37]. The statistical significance threshold was set at *p* < 0.05 with a false discovery rate (FDR) correction.

#### Machine learning analysis

We used brain areas which had significantly disrupted FC with NAc as potential features and examined their ability to discriminate patients with recurrent MDDs from healthy controls by using a support vector machine (SVM) in Matlab. SVM could handle data in a high-dimensional space and create a hyperplane that could best classify a new target into predefined categories. A Grid search method was used to obtain optimal values of hyperparameters (such as parameter C and Gamma) of a model. A fivefold cross-validation method was employed to assess the generalizability of classifier models. We trained those models with a Gaussian kernel in 80% of participants and evaluated the models’ performance on the left-out data in the “testing phase” for each fold. This method is good at handling high-dimensional data with optimal boundaries constructed and misclassification error minimized and is less likely to overfitting of the data [38, 39]. Finally, accuracy was calculated based on the results of cross-validation:

$${\mathrm{Accuracy}} = \left({\mathrm{TP}}\, +\, {\mathrm{TN}}\right)/\left({\mathrm{TP}}\, +\, {\mathrm{FN}}\, + \,{\mathrm{TN}} \,+ \,{\mathrm{FP}}\right)$$

where TP and TN represent the number of patients and controls correctly predicted, respectively; FP represents the number of controls classified as patients and FN represents the number of patients classified as controls.

## Results

### Meta-analysis

#### Characteristics of eligible studies

Twelve studies involving 1068 subjects (593 patients and 475 healthy controls) were included in the meta-analysis. Figure S2 shows the flow diagram of the identified, included, and excluded studies. The characteristics of the included studies are shown in the Supplementary Materials (Table S2). The majority of patients recruited in these studies were patients with recurrent MDD and aged 18–60 years old. Two studies have focused on adolescent (12–19 years) and older adults (>60 years) patients with MDD [18, 40]. Nine of the studies selected bilateral NAc as seeds, and the other three selected bilateral inferior VS as seeds. Symptomatic severity was not evaluated by HAMD in two studies, so they were not included in the meta-regression analysis [18, 41].

#### Group differences and meta-regression analyses

**Fig. 1:**
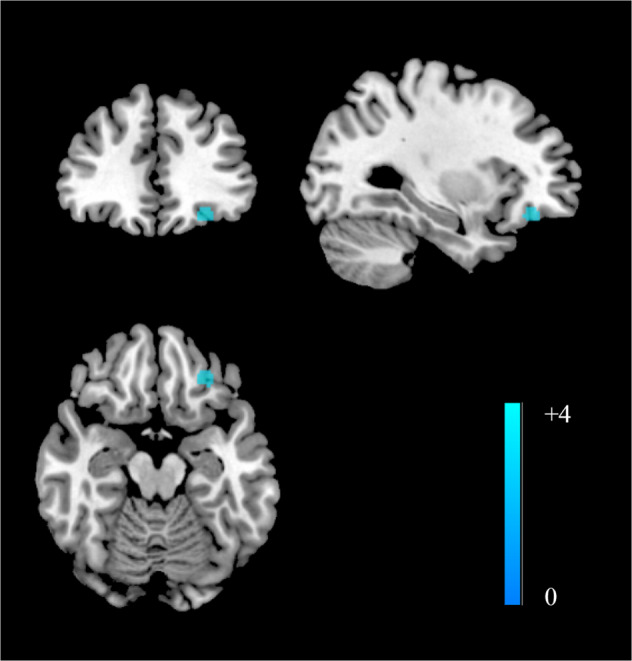
Group differences of meta-analysis. Compared with healthy controls, patients with recurrent MDD exhibited decreased NAc functional connectivity in the left ventromedial prefrontal cortex.

As shown in Fig. 1, compared with healthy controls, patients with recurrent MDD had decreased FC between the NAc and left ventromedial prefrontal area (peak MNI = −28, 36, −14, *z* = −4.089, *p* < 0.001, 59 voxels). No increased FCs were reported. Slight but not significant heterogeneity was found in this area (*I*^*2*^ = 0.95%, *p* = 0.968). No obvious publication bias was observed in the funnel plot (Supplementary Materials and Fig. S3). When we adopted a relatively lenient threshold at *p* < 0.01, significant results included the left ventromedial prefrontal area (peak MNI = −28, 36, −14, *z* = −4.089, *p* < 0.001, 205 voxels) and right ACC (peak MNI = 4, 36, −6, *z* = −2.519, *p* = 0.006, 12 voxels).

The meta-regression analysis did not find any significant effects of depressive severity or sex ratio on NAc FC maps.

Our supplementary analysis for studies only focused on the NAc showed no significantly positive results at a threshold of *p* < 0.001(uncorrected). When threshold was set at *p* < 0.01, decreased FC between the NAc and left ventromedial prefrontal area (peak MNI = −28, 36, −14, *z* = −3.219, *p* < 0.001, 115 voxels) was observed.

### Mega-analysis

#### Group comparisons

The demographic and clinical characteristics of the included subjects are shown in Table 1. No significant differences were reported between patients with recurrent MDD and healthy controls in age and sex ratio. Compared with healthy controls, patients with recurrent MDD exhibited lower education levels.

**Table 1:** Demographic and clinical characteristics of the participants included in the analysis.

	Recurrent MDDs		HCs		Group comparisons
	Mean	SD	Mean	SD	*p*
Age	35.35	12.43	37.14	3.84	0.132^a^
Education	11.78	3.35	13.35	3.84	<0.0001^a^
Duration	88.44	85.22	-	-	-
HAMD	21.38	5.15	-	-	-
Sex	Male	Female	Male	Female	*p*
	81	105	180	285	0.288^b^

*HAMD* Hamilton rating scale for depression, *HC* healthy control, *MDD* major depressive disorder, *SD* standard deviation.

^a^The *p* value was obtained by two-sample *t*-tests.

^b^The *p* value was obtained by a chi-square test.

**Fig. 2:**
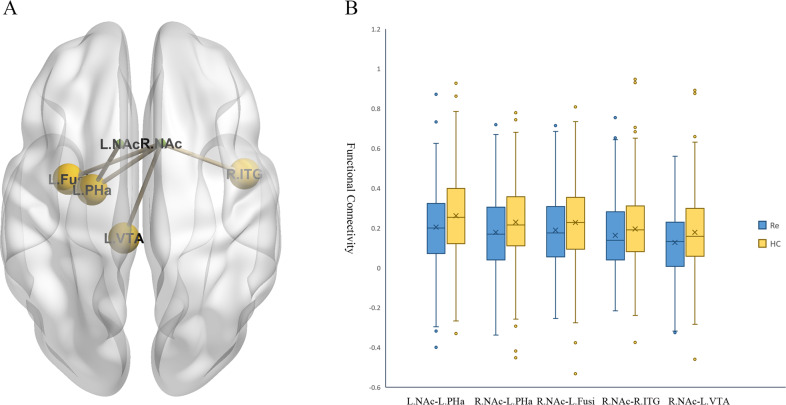
Results of mega-analysis. **A** The significant between-group differences in the NAc functional connectivity. **B** The box figures show the distribution of significantly disrupted bilateral NAc functional connectivity in the group comparison. Fusi fusiform gyrus, HC healthy control, L left, NAc nucleus accumbens, PHa parahippocampal gyrus, R right, Re recurrent major depressive disorder, ITG inferior temporal gyrus, VTA ventral tegmental area.

As shown in Table 2 and Fig. 2, compared with healthy controls, patients with recurrent MDD mainly exhibited generally decreased bilateral NAc FC in the reward network, including the left hippocampus/parahippocampal gyrus (left NAc seed: *t* = −3.903, *p* < 0.001, *d* = −0.340; right NAc seed: *t* = −3.705, *p* < 0.001, *d* = −0.324) and left VTA (right NAc seed: *t* = −3.342, *p* = 0.028, *d* = −0.292). A region within the DMN (right lateral temporal cortex, *t* = −3.347, *p* = 0.037, *d* = −0.292) and a region involved in visual processing (left fusiform gyrus, *t* = −4.507, *p* < 0.001, *d* = −0.393) were also survived multiple comparison correction (The uncorrected results were showed in Supplementary Materials Table S3). No significant associations were found between bilateral NAc FC maps and HAMD scores or subscores in the patients.

**Table 2:** The significant between-group differences in functional connectivity for the NAc (*p* < 0.05, FDR-corrected).

Cluster Location	network	MNI			*t*	*d*	*p*
		x	y	z			
*Seed: Left NAc*							
L_Parahippocampal gyrus, anterior division	Reward network	−21.68	−9.28	−30.7	−3.903	−0.340	<0.001
*Seed: Right NAc*							
R_Inferior Temporal Gyrus, anterior division	DMN	46.31	−2.16	−41.18	−3.347	−0.292	0.037
L_Parahippocampal Gyrus, anterior division	Reward network	−21.68	−9.28	−30.7	−3.705	−0.324	<0.001
L_Ventral tegmental area	Reward network	−7.49	−30.76	−33.99	−3.342	−0.292	0.028
L_Fusiform cortex, anterior division	Visual network	−32.3	−4.53	−41.6	−4.507	−0.393	<0.001

*DMN* default mode network, *FDR* false discovery rate, *MNI* the Montreal Neurological Institute space coordinates, *NAc* nucleus accumbens.

#### Distinguishing patients with recurrent MDD from healthy controls

**Fig. 3:**
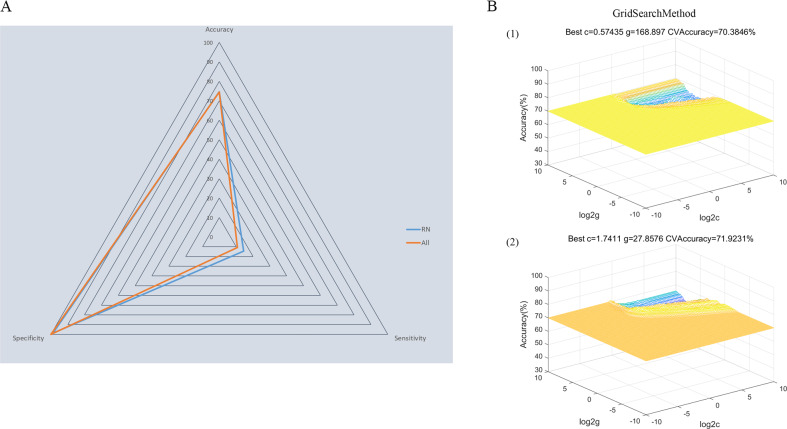
Visualization of classification by SVM analyses. **A** The accuracy, sensitivity, and specificity of the classifications of patients with recurrent MDD versus HCs using all significant NAc FCs as features or using significant NAc FCs within the reward network as features. **B** Parameter selection result for training set when using (1) all significant NAc FCs as features and (2) using significant NAc FCs within the reward network as features. FC functional connectivity, HC healthy controls, MDD major depressive disorder, NAc nucleus accumbens, SVM support vector machine.

Significantly abnormal bilateral NAc FCs returned by the former step were used as potential features for subsequent SVM analysis. As shown in Fig. 3, the combination of all significant NAc FCs as features could discriminate patients with recurrent MDD from healthy controls with an accuracy of 74.5%; when using significant NAc FCs within the reward network as features, the discriminative accuracy was 74.7%. The specificities of the two strategies were high (100% and 98.7%, respectively), whereas neither of the sensitivities were more than 20%.

## Discussion

Using a meta-analysis and a large MDD sample for mega-analysis, we found decreased FCs within the NAc-based reward system in patients with recurrent MDD compared with healthy controls as predicted. In addition, reduced NAc-DMN FCs were prominent. No significantly increased FCs between the NAc and other brain regions were reported. Specifically, the combinations of the disrupted NAc FCs within the reward network could discriminate patients with recurrent MDD from healthy controls with an optimal accuracy of 74.7%. Though the sensitivity was not satisfactory, the high specificity could ensure the correct identification of healthy individuals.

This study has two main novel aspects. First, we used a new coordinate-based meta-analysis method, namely, SDM-PSI, to statistically summarize up-to-date voxel-based rs-fMRI studies that focused on the NAc and reward network in patients with MDD. This method benefits from the control of family-wise error rate via subject-based permutation test and the control of bias introduced by exclusion of studies with non-significant results and unknown statistics via multiple imputations algorithms-based MetaNSUE method [29]. Second, we used a large rs-fMRI database of MDD from 25 sites in China to cross-validate the findings of the meta-analysis. These imaging data were all preprocessed in the same protocol with the DPARSF software and thus eliminating analytic heterogeneity. Furthermore, we employed SVM to test the potential of the disrupted NAc-based FC map as a biomarker of MDD in this database. The relatively high accuracy of our SVM analyses indicated the important roles of the reward network and its inter-network connectivity with the DMN in the pathophysiology of MDD, and may have promising clinical implications to provide helpful scaffolding in promoting objective diagnostic tools for MDD and developing individualized treatment.

The most robust finding in this study was decreased FC within the reward system, which was observed both in the meta-analysis and mega-analysis. The ACC, mPFC, and hippocampus/parahippocampal gyrus are important hubs in the reward circuit and play critical roles in reward motivation and reappraisal, emotional regulation, and reasoning along with the cognitive control network [24, 42, 43]. Histopathological studies have reported neuron size reduction and glia loss in the ACC and mPFC [44–46]. Mounting evidence from the fMRI studies has implicated alterations of these regions in patients with different ages [47–50]. Chen et al. found altered variability in the dynamic FC between the inferior VS and mPFC in patients with MDD compared with healthy controls and patients with bipolar disorder II, which indicates an MDD-specific alteration [51]. Notably, a negative association between the NAc-subgenual ACC intrinsic FC and anhedonia severity has been reported in adolescent patients with MDD [18]. Anhedonia, which reflects deficits in pleasure feeling and reward processing, is a core symptom of MDD and even persists after treatment [52, 53]. A previous meta-analysis reported that decreased activation in the left ACC was associated with consummatory anhedonia in patients with MDD [54]. Similarly, Rzepa et al. observed blunted neural responses in the ACC and vmPFC during the consummatory phase of rewarding stimuli in young people at risk of depression [55].

It is well established that the hippocampus/parahippocampal gyrus and VTA have close connections with the NAc. The hippocampus receives and sends inputs to the NAc and plays critical and complex roles in the processing of reward valence [56]. The VTA is a heterogeneous brain region, which has dopaminergic and GABAergic projections to the NAc [56]. Previous task fMRI studies found decreased FC between the VTA and striatum in patients with MDD during reward outcome in monetary instrumental learning task compared to healthy individuals, which indicated reward-related deficit in MDD and the underlying disrupted VTA-NAc circuit [57]. Shi et al. reported increased resting-state FCs between the bilateral VTA and ventral striatum in MDD compared to healthy controls using an ROI-to-ROI analysis, but these results were not survived correction for multiple comparisons [58]. Using the same ROI-based method, Wang et al. observed reduced resting-state NAc FCs in the bilateral hippocampus in MDD [25]. Intriguingly, our meta-analysis for all up-to-date NAc-based whole-brain resting-state fMRI studies did not find any significant positive results in the subcortical areas within the reward network, which instead were the main findings in the mega-analysis. Methodology differences may contribute to the inconsistence. In addition to different data preprocessing and analysis operations and diverse multiple comparison correction methods, disparities in NAc definition may be one of the meaningful factors. According to the results of supplementary meta-analysis, the inclusion of ventral striatum in the meta-analysis did not seem to contribute to the heterogeneity, instead, the subregions of NAc are worth noting. The NAc has a core-like and a shell-like subdivision, and the two parts have connections with different regions associated with different reward-related functions [59, 60]. Given the coordinates of bilateral NAc in the Harvard-Oxford Atlas used in the mega-analysis and those used in the meta-analysis, findings from the meta-analysis were more likely to present an abnormal NAc shell-like subdivision-based FC profiles in patients with recurrent MDD. Albeit the disparities, the combination of meta-analysis and mega-analysis in this study highlighted the disrupted NAc-centered reward network in MDD.

Abnormal inter-network connectivity between the DMN and NAc (reward network) in the recurrent MDDs was another prominent result in this study. The ventral mPFC, the significantly positive result in the meta-analysis, was regarded as an important part of the DMN [61], and the DMN was considered to be engaged in reward processing. For instance, Olivo and colleagues reported that connectivity in the DMN (such as lateral temporal regions) was related to reward sensitivity [62]. Altered activation of the DMN was observed in effort avoidance behavior, which suggested that the DMN activity might be associated with reward processing that predicts effort selection [63]. The reward network-DMN FC deficits showed in our study indirectly corroborated previous studies. Using the precuneus/posterior cingulate gyrus as a seed, researchers found decreased DMN-bilateral caudate connectivity in early depression [64]. Hwang et al. [65] found increased FC between the DMN and ventral striatum in two cohorts of subthreshold depression and the value of DMN-ventral striatum FC was positively related to scores of depressive symptomatology (measured by the Center for Epidemiologic Studies Depression Scale). This result was regarded as compensation for the lowered reward function in patients with subthreshold depression. Taken together, our study and previous studies demonstrated disrupted FC between the reward network and DMN in MDD, and such abnormality may change at different stages of depression.

Unexpectedly, we found altered FC between the NAc and fusiform gyrus (a region adjoins hippocampus/parahippocampal gyrus and involves in visual pathways of recognition [66]) in the mega-analysis. On the one hand, some studies have indicated a possible role of the visual system in patients with MDD. Altered blood-oxygenation-level-dependent signals in the occipital lobes were found during the facial expression tasks [67], working memory tasks [68], and reward tasks [69]. Reduced nodal centralities in the occipital regions were documented in a study that investigated the topological organization of brain networks during the resting state in patients with MDD [70]. Processing emotion- or reward-related visual stimuli in the task and eyes-open status at rest may provide explanations for these findings [69, 71–73]. In addition, mixed medication treatments and different antidepressant responses in patient with recurrent MDD may affect the activation of the occipital cortex. For example, compared with non-responders, remitters had a more obvious decrease in activation in the occipital cortex following escitalopram treatment [74]. On the other hand, a significantly positive result seen in the fusiform gyrus in our mega-analysis may be a result of the extended effect of the hippocampus/parahippocampal gyrus. Thus, it is still unclear whether inter-network connectivities between the reward network and visual system play roles in MDD due to limited evidence.

Several limitations of this study should be acknowledged. First, most studies included in the meta-analysis and all patients recruited in the mega-analysis were from China. Thus, the results should be taken with caution when generalizing to other populations. Data from other ethnicity/culture (such as the UK biobank) should be analyzed and compared with our results. Similarly, combined data from other international data-sharing consortiums will help explore MDD subtypes (clinical-symptom based or neurophysiological based). Second, medication details are not available in our study and thus may confound the results. As previously mentioned, antidepressants and response disparities may have different effects on the brain function [74, 75]. Third, permutation tests used in the SDM-PSI analysis were not entirely free from bias. Images recreated from peak *t* values could not fully duplicate raw images [29]. Finally, results of associations between disrupted NAc-based reward network FC and HAMD scores were mixed in the meta-analysis and mega-analysis. Whether illness severity contributes to the abnormalities remains unknown. Future studies need to focus on specific symptoms (such as anhedonia) instead of using general rating scales.

In summary, by combining a meta-analysis and a large MDD sample for mega-analysis, we confirmed the critical role of NAc-based reward system in MDD. We identified decreased FC in the NAc-based reward network in patients with recurrent MDD. Disrupted inter-network connectivity between the reward network and DMN may also have contributed to the pathophysiological mechanisms of MDD. Moreover, a combination of abnormal NAc FCs in the reward network can serve as potential brain-based biomarkers for individual diagnosis to differentiate patients with recurrent MDD from healthy controls.

## Supplementary information

Supplementary Materials
