# Supplementary material for: Reduced nucleus accumbens functional connectivity in reward network and default mode network in patients with recurrent major depressive disorder
Source: Transl Psychiatry. 2022 Jun 6;12:236. doi: 10.1038/s41398-022-01995-x (PMC9170720; doi:10.1038/s41398-022-01995-x)
Supplement: Supplementary file 1 — Supplementary Materials [file 41398_2022_1995_MOESM1_ESM.docx]

Data preprocessing

After slice-timing and head motion correction, participants with more than 2 mm maximal translation and 2° maximal rotation were excluded. Then, the images were normalized to Montreal Neurological Institute space and resampled to 3 × 3 × 3 mm3 voxels. Finally, the images were bandpass-filtered (0.01–0.08 Hz) and linearly detrended. Several variances were removed, including 24-parameters acquired via rigid body correction, signal from a ventricular region-of-interest, and signal from a region centered in the white matter. Additionally, mean framewise displacement (FD) was applied to address the residual effects of motion as a covariate in group analyses. Scrubbing (removing time points with FD > 0.2mm) was used as an aggressive head motion control strategy. We did not remove the global signal since it is still controversial to regress out it in the resting-state functional magnetic resonance imaging (fMRI) field [1,2].

Table S1 fMRI data acquisition parameters of each study site.

| Cohort number | Scanner | Receive (coil) | TR (ms) | TE (ms) | Flip Angle (︒) | Thickness/gap | Slice number | Time points | Voxel size | FOV |
| --- | --- | --- | --- | --- | --- | --- | --- | --- | --- | --- |
| 1 | Siemens Tim Trio 3T | 32 channel | 2000 | 30 | 90 | 4.0mm/0.8mm | 30 | 210 | 3.28 × 3.28 × 4.80 | 210 × 210 |
| 2 | Philips Achieva 3T | 8 channel | 2000 | 30 | 90 | 4.0mm/0 mm | 37 | 200 | 1.67 × 1.67 × 4.00 | 240 × 240 |
| 3 | Siemens Magnetom Symphony scanner 1.5 T | 16 channel | 2000 | 40 | 90 | 5.0mm/1.25mm | 26 | 150 | 3.75 × 3.75 × 6.25 | 240 × 240 |
| 4 | Siemens Skyra 3T | 32 channel | 2500 | 25 | 90 | 3.5mm/0mm | 39 | 200 | 3.75 × 3.75 × 3.50 | 240 × 240 |
| 5 | GE Signa 3T | 32 channel | 3000 | 30 | 90 | 5.0mm/0mm | 22 | 100 | 3.75 × 3.75 × 5.00 | 240 × 240 |
| 6 | Siemens Tim Trio 3T | 32 channel | 2000 | 30 | 70 | 4mm/0mm | 33 | 180 | 3.59 × 3.59 × 4.00 | 230 × 230 |
| 7 | GE discovery MR750 | 8 channel | 2000 | 30 | 90 | 3.2mm/0mm | 37 | 184 | 2.29 × 2.29 × 3.20 | 220 × 220 |
| 8 | GE Signa 3T | 8 channel | 2000 | 30 | 90 | 3.0mm/0mm | 35 | 200 | 3.75 × 3.75 × 3.00 | 240 × 240 |
| 9 | GE Discovery MR750 3.0T | 8 channel | 2000 | 25 | 90 | 3.0mm/1.0 mm | 35 | 200 | 3.75 × 3.75 × 4.00 | 240 × 240 |
| 10 | Siemens Tim Trio 3T | 32 channel | 2000 | 30 | 90 | 3.0mm/1.52mm | 32 | 212 | 3.75 × 3.75 × 4.52 | 240 × 240 |
| 11 | GE Signa 3T | 8 channel | 2000 | 30 | 90 | 5mm | 33 | 200 | 3.75 × 3.75 × 5.00 | 240 × 240 |
| 12 | GE Signa 3T | 8 channel | 2000 | 30 | 90 | 5mm | 33 | 240 | 3.75 × 3.75 × 4.00 | 240 × 240 |
| 13 | GE Excite 1.5T | 16 channel | 2500 | 35 | 90 | 4mm/0mm | 36 | 150 | 4.00 × 4.00 × 4.00 | 256 × 256 |
| 14 | Siemens Tim Trio 3T | 32 channel | 2500 | 25 | 90 | 3.5mm/0mm | 39 | 200 | 3.75 × 3.75 × 3.50 | 240 × 240 |
| 15 | Siemens Verio 3.0T MRI | 12 channel | 2000 | 25 | 90 | 4mm/0mm | 36 | 240 | 3.75 × 3.75 × 4.00 | 240 × 240 |
| 16 | GE Signa 3T | 8 channel | 2000 | 30 | 90 | 5mm/0mm | 30 | 200 | 3.75 × 3.75 × 5.00 | 240 × 240 |
| 17 | GE Signa 3T | 8 channel | 2000 | 40 | 90 | 4.0mm/0mm | 33 | 240 | 3.75 × 3.75 × 4.00 | 240 × 240 |
| 18 | Philips Achieva 3.0 T scanner | 8 channel | 2000 | 35 | 90 | 5.0mm/1.0 mm | 24 | 200 | 1.67 × 1.67 × 6.00 | 240 × 240 |
| 19 | GE Signa 3T | 8 channel | 2000 | 22.5 | 30 | 4.0mm/0.6 mm | 33 | 240 | 3.44 × 3.44 × 4.60 | 220 × 220 |
| 20 | Siemens Tim Trio 3T | 12 channel | 2000 | 30 | 90 | 3.0mm/1.0mm | 32 | 242 | 3.44 × 3.44 × 4.00 | 220 × 220 |
| 21 | Siemens Tim Trio 3T | 32 channel | 2000 | 30 | 90 | 3.5mm/0.7mm | 33 | 240 | 3.12 × 3.12 × 4.20 | 200 × 200 |
| 22 | Philips Gyroscan Achieva 3.0T | 32 channel | 2000 | 30 | 90 | 4.0mm/0mm | 36 | 250 | 1.67 × 1.67 × 4.00 | 240 × 240 |
| 23 | Philips Achieva 3.0T TX | 8 channal | 2000 | 30 | 90 | 4.0mm/0mm | 38 | 240 | 3.75 × 3.75 × 4.00 | 240 × 240 |
| 24 | GE Signa 1.5T | 8 channel | 2000 | 40 | 90 | 5mm/1mm | 24 | 160 | 3.75 × 3.75 × 6.00 | 240 × 240 |
| 25 | Siemens Verio 3T | 12 channel | 2000 | 25 | 90 | 4.0mm/0mm | 36 | 240 | 3.75 × 3.75 × 4.00 | 240 × 240 |

Abbreviations: FOV, field of vision; TE, time of echo; TR, time of repetition.

Table S2 Characteristics of studies included in meta-analysis

| Study | Type of analysis | Patients | | | | | | Healthy controls | | | |
| --- | --- | --- | --- | --- | --- | --- | --- | --- | --- | --- | --- |
|  |  | Diagnosis | N1 | Age | Males% | Education | Severity | N2 | Age | Males% | Education |
| Wang et al. 2020[3] | Whole-brain seed-based (VSi) | MDD (DSM-IV) | 29 | 34.0 ± 11.7 | 51.7 | 11.6 ± 2.9 | 22.7 ± 4.4 (HAMD-17) | 57 | 36.0 ± 11.6 | 56.1 | 11.5 ± 2.2 |
| Tadayonnejad et al. 2014[4] | Whole-brain ROI-wise (NAc) | MDD (DSM-IV) | 10 | 62.9 ± 2.5 (>60) | 40 | 14.5 ± 2.6 | 19.6 ± 3.5 (HAMD-17) | 15 | 71.7 ± 6.8 | 46.6 | 14.3 ± 2.1 |
| Meng et al. 2021[5] | Whole-brain seed-based (NAc) | MDD (DSM-IV) | 30 | 28.83 ± 10.15 | 26.7 | - | 16.43 ± 6.03 (HAMD-24) | 18 | 27.11 ± 6.34 | 33.3 | - |
| Kerestes et al. 2014[6] | Whole-brain seed-based (NAc) | MDD (DSM-IV) | 21 | 19.3 ± 2.5 | 48 | 12.1 ± 1.9 | 33.7 (MADRS) | 21 | 19.2 ± 2.3 | 48 | 13 ± 1.6 |
| Gong et al. 2017[7] | Whole-brain seed-based (NAc) | MDD (DSM-IV) | 53 | 39.81 ± 11.64 | 49 | 9.98 ± 4.00 | 22.69 ± 4.69 (HAMD-17) | 37 | 42.19 ± 11.49 | 56.8 | 11.54 ± 4.07 |
| Ang et al. 2020^†^[8] | Whole-brain seed-based (NAc) | MDD (DSM-IV) | 16 | 37.0 ± 14.6 | 37.5 | 15.6 ± 2.0 | 18.5 ± 4.0 (HAMD-17) | 38 | 37.4 ± 14.9 | 39.5 | 15.6 ± 4.5 |
|  |  |  | 22 | 39.4 ± 15.1 | 22.7 | 14.6 ± 3.0 | 19.2 ± 4.6 |  |  |  |  |
|  |  |  | 25 | 42.1 ± 11.9 | 36 | 15.4 ± 2.6 | 18.3 ± 4.6 |  |  |  |  |
|  |  |  | 24 | 40.0 ± 14.5 | 45.8 | 15.4 ± 2.5 | 18.7 ± 3.2 |  |  |  |  |
| Gong et al. 2016[9] | Whole-brain seed-based (NAc) | MDD (DSM-IV) | 75 | 40.41 ± 11.59 | 44 | 9.77 ± 3.59 | 21.29 ± 5.12 (HAMD-17) | 42 | 41.31 ± 11.65 | 54.8 | 11.67 ± 4.27 |
| Wang et al. 2019[10] | Whole-brain seed-based (VSi) | MDD (DSM-IV) | 23 | 29.7 ± 5.8 | 47.8 | 14.3 ± 3.2 | 30.5 ± 5.4 (HAMD-24) | 23 | 29.3 ± 5.9 | 47.8 | 15.6 ± 1.8 |
| Liu et al. 2021[11] | Whole-brain seed-based (NAc) | MDD (DSM-IV) | 23 | 31.22 ± 5.7 | 56.5 | 14.91 ± 3.22 | 13.13 ± 6.96 (HAMD-17) | 28 | 29.57 ± 5.8 | 57.1 | 15.25 ± 2.55 |
| Gabbay et al. 2013[12] | Whole-brain seed-based (NAc) | MDD (DSM-IV) | 21 | 17.1 ± 2.5 | 42.8 | - | (CDRS-R) | 21 | 16.3 ± 1.4 | 42.8 | - |
| Chen et al. 2020[13] | Whole-brain seed-based (VSi) | MDD (DSM-V) | 140 | 27.68 ± 11.52 | 40 | 12.91 ± 3.2 | 25.27 ± 6.06 (HAMD-24) | 132 | 29.09 ± 8.8 | 46.2 | 15.39 ± 3.4 |
| Hou et al. 2017^‡^[14] | Whole-brain seed-based (NAc) | MDD (DSM-V) | 44 | 48.75 ± 13.31 | 27.3 | 9.27 ± 4.00 | 28.93 ± 7.35 (HAMD-24) | 43 | 48.77 ± 17.52 | 46.5 | 11.02 ± 3.95 |
|  |  |  | 37 | 47.11 ± 15.86 | 27 | 9.49 ± 4.32 | 29.95 ± 6.33 |  |  |  |  |

Abbreviations: CDRS, Children’s Depression Rating Scale-Revised; DSM, Diagnostic and Statistical Manual of Mental Disorders; HAMD, Hamilton rating scale for depression; MDD, major depressive disorder; NAc, nucleus accubens; VSi, inferior ventral striatum.

Notes: †, In this study, four patient groups, sertraline responders and nonresponders and bupropion responders and nonresponders, were compared with controls respectively.

‡, This study divided patients into treatment-responsive depression and non-responding depression.

Table S3 The significant between-group differences in functional connectivity for the NAc (p <0.05, uncorrected)

| Cluster Location | network | MNI |  |  | *t* | *d* | *p* |
| --- | --- | --- | --- | --- | --- | --- | --- |
|  |  | x | y | z |  |  |  |
| *Seed: Left NAc* |  |  |  |  |  |  |  |
| R_ Medial Frontal Cortex | DMN/reward network | 5.51 | 43.41 | -18.24 | -2.788 | -2.594 | 0.034 |
| R_Angular Gyrus | DMN | 52.15 | -51.69 | 32.16 | -2.840 | -0.248 | 0.005 |
| L_Inferior Temporal Gyrus, anterior division | DMN | -47.97 | -5.1 | -39.12 | -2.587 | -0.226 | 0.010 |
| L_Angular Gyrus | DMN | -50.45 | -55.74 | 29.3 | -2.501 | -0.218 | 0.013 |
| R_Planum Temporale | DMN | 54.84 | -25.33 | 12.39 | -2.357 | -0.206 | 0.019 |
| L_Superior Parietal Lobule | DMN | -29.28 | -49.4 | 57.63 | -2.284 | -0.199 | 0.023 |
| R_Inferior Temporal Gyrus, anterior division | DMN | 46.31 | -2.16 | -41.18 | -2.201 | -0.192 | 0.028 |
| R_Amygdala | Reward network | 22.77 | -3.69 | -17.91 | -3.056 | -2.784 | 0.023 |
| L_Parahippocampal Gyrus, anterior division | Reward network | -21.68 | -9.28 | -30.7 | -3.903 | -0.340 | < 0.001 |
| R_Parahippocampal Gyrus, posterior division | Reward network | 22.95 | -30.25 | -16.98 | -1.995 | -0.176 | 0.046 |
| L_Insular Cortex | Sensorimotor network | -36.42 | 1.01 | 0.16 | -2.188 | -0.191 | 0.029 |
| L_Supplementary Motor Cortex | Sensorimotor network | -5.79 | -2.67 | 56.3 | -2.661 | -0.234 | 0.008 |
| L_Central Opercular Cortex | Sensorimotor network | -48.03 | -8.28 | 11.64 | -2.418 | -0.227 | 0.016 |
| R_Parietal Operculum Cortex | Sensorimotor network | 48.86 | -27.69 | 21.65 | -2.483 | -0.216 | 0.013 |
| L_Lateral Occipital Cortex, superior division | Visual system | -32.05 | -72.77 | 37.99 | -2.804 | -0.245 | 0.005 |
| L_Fusiform Cortex, anterior division | Visual system | -32.3 | -4.53 | -41.6 | -4.280 | -3.797 | 0.005 |
| R_Fusiform Cortex, anterior division | Visual system | 30.87 | -2.55 | -42.28 | -2.569 | -0.224 | 0.010 |
| *Seed: Right NAc* |  |  |  |  |  |  |  |
| R_Inferior Temporal Gyrus, anterior division | DMN | 46.31 | -2.16 | -41.18 | -3.347 | -0.292 | 0.001 |
| R_Middle Temporal Gyrus, anterior division | DMN | 57.86 | -1.74 | -24.52 | -2.353 | -0.210 | 0.019 |
| L_Middle Temporal Gyrus, temporooccipital part | DMN | -57.4 | -52.7 | 0.87 | -2.242 | -0.195 | 0.025 |
| R_Temporal Pole | DMN | 40.99 | 12.93 | -29.31 | -2.163 | -0.189 | 0.031 |
| L_Planum Temporale | DMN | -52.64 | -29.69 | 10.8 | -2.133 | -0.186 | 0.033 |
| L_Parahippocampal Gyrus, anterior division | Reward network | -21.68 | -9.28 | -30.7 | -3.705 | -0.324 | < 0.001 |
| L_Ventral tegmental area | Reward network | -7.49 | -30.76 | -33.99 | -3.342 | -0.292 | 0.001 |
| R_Parahippocampal Gyrus, anterior divisio | Reward network | 22.59 | -8.04 | -30.63 | -2.804 | -0.245 | 0.005 |
| L_Subcallosal Cortex | Reward network | -5.7 | 20.6 | -15.68 | -2.490 | -0.217 | 0.013 |
| R_Subcallosal Cortex | Reward network | 5.66 | 20.42 | -15.9 | -2.255 | -0.197 | 0.024 |
| R_Amygdala | Reward network | 22.77 | -3.69 | -17.91 | -2.126 | -0.185 | 0.034 |
| R_Hippocampus | Reward network | 26.5 | -20.99 | -14.07 | -2.106 | -0.184 | 0.036 |
| L_Insular Cortex | Sensorimotor network | -36.42 | 1.01 | 0.16 | -2.174 | -0.190 | 0.030 |
| R_Insular Cortex | Sensorimotor network | 37.5 | 2.65 | -0.17 | -2.214 | -0.193 | 0.027 |
| L_Parietal Operculum Cortex | Sensorimotor network | -48.4 | -31.53 | 20.3 | -2.339 | -0.208 | 0.020 |
| R_Parietal Operculum Cortex | Sensorimotor network | 48.86 | -27.69 | 21.65 | -2.291 | -0.202 | 0.022 |
| L_Fusiform Cortex, anterior division | Visual system | -32.3 | -4.53 | -41.6 | -4.507 | -0.393 | < 0.001 |
| L_Fusiform Cortex, posterior division | Visual system | -36.02 | -29.45 | -25.04 | -2.341 | -0.204 | 0.020 |

Abbreviations: DMN, default mode network; MNI, the Montreal Neurological Institute space coordinates; NAc, nucleus accumbens.

Figure S1 Sample selection


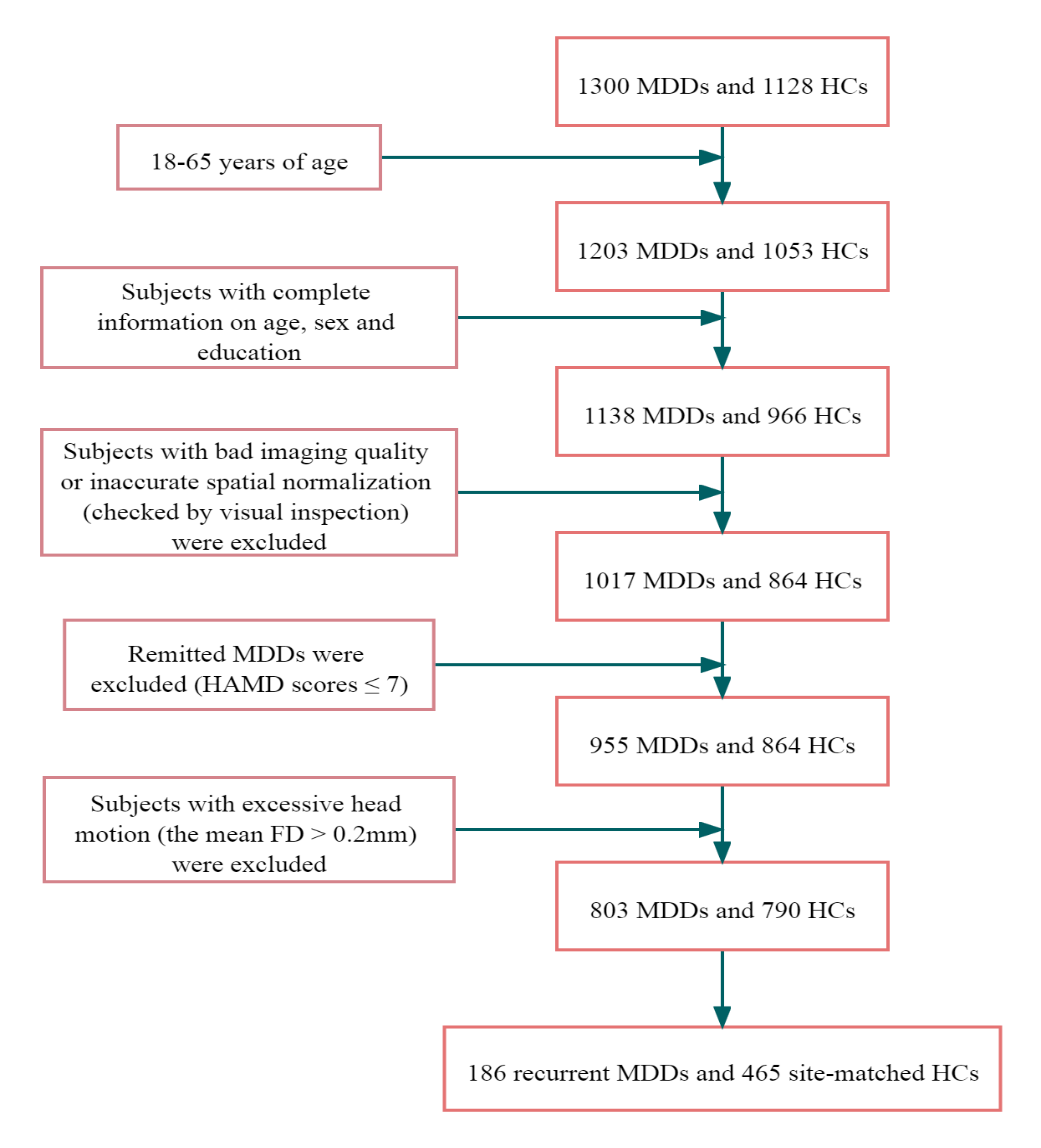


Figure S2 Search strategy


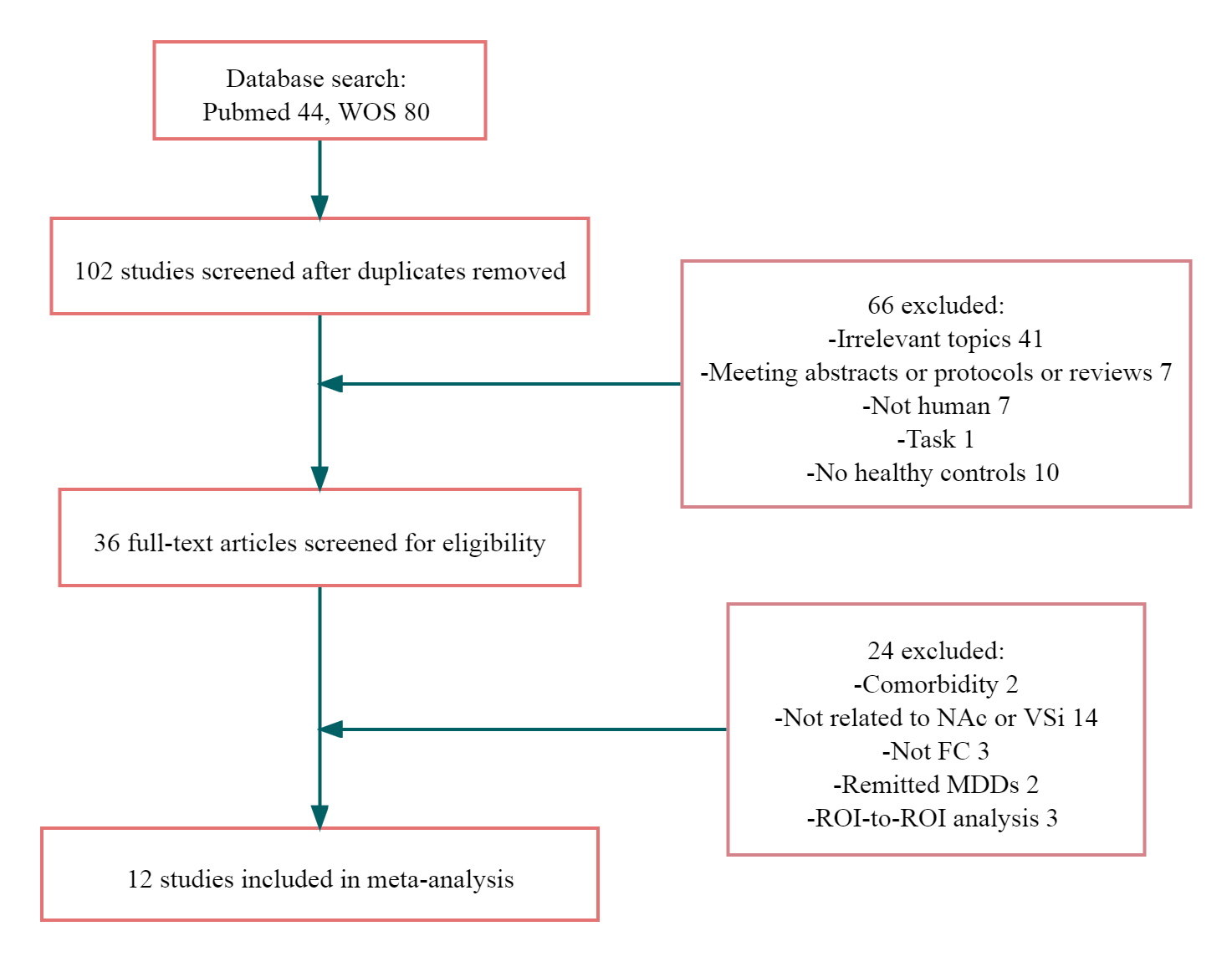


Figure S3 Funnel plot of meta-analysis


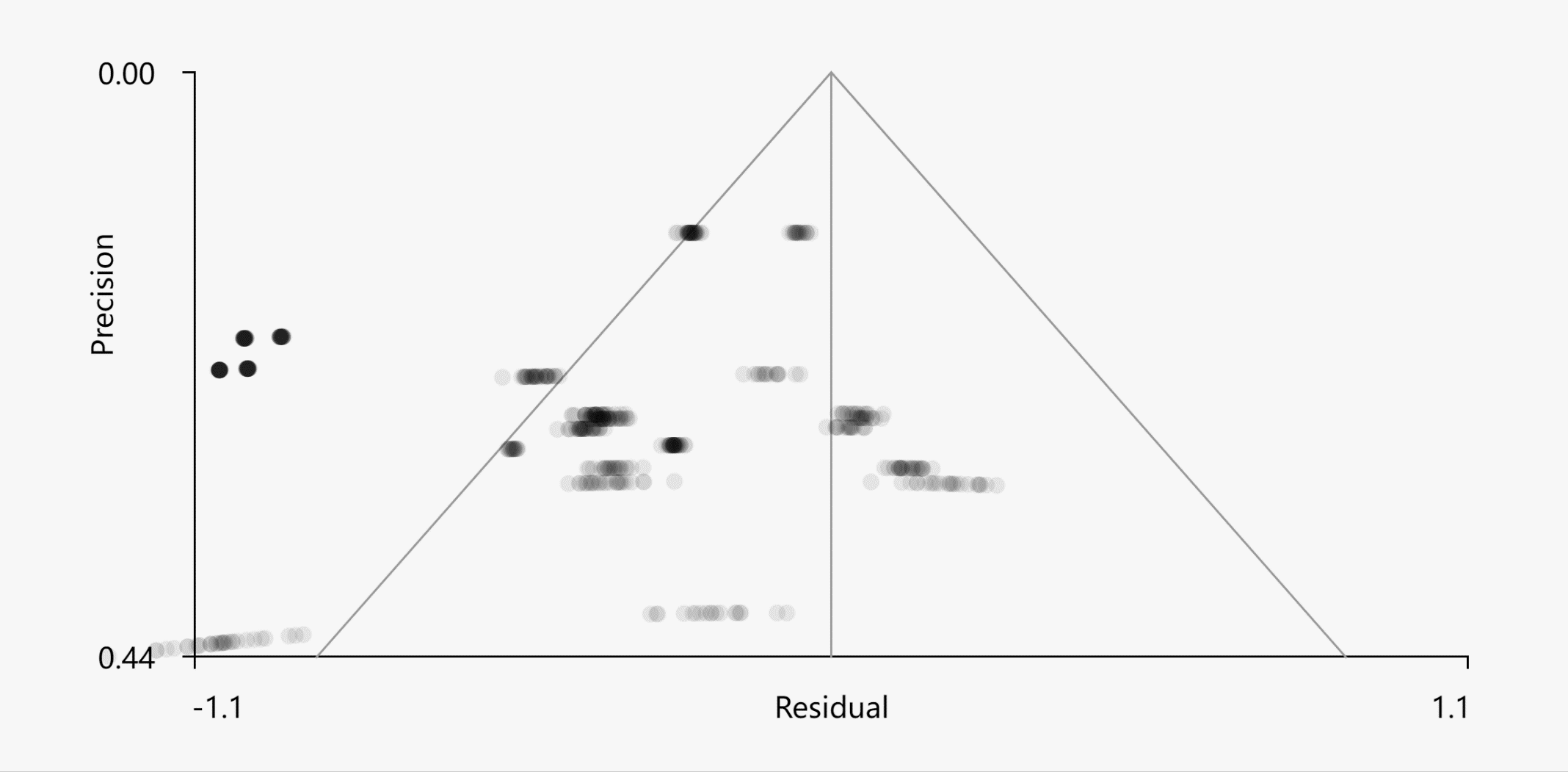


References:

[1] Fox, M. D., Snyder, A. Z., Vincent, J. L., Corbetta, M., Van Essen, D. C.,Raichle, M. E. The human brain is intrinsically organized into dynamic, anticorrelated functional networks. *Proc Natl Acad Sci U S A* https://doi.org:10.1073/pnas.0504136102 (2005).

[2] Murphy, K., Birn, R. M., Handwerker, D. A., Jones, T. B.,Bandettini, P. A. The impact of global signal regression on resting state correlations: are anti-correlated networks introduced? *Neuroimage* https://doi.org:10.1016/j.neuroimage.2008.09.036 (2009).

[3] Wang, L., Li, F., Mitchell, P. B., Wang, C. Y.,Si, T. M. Striatal Resting-State Connectivity Abnormalities Associated With Different Clinical Stages of Major Depressive Disorder. *J Clin Psychiatry* https://doi.org:10.4088/JCP.19m12790 (2020).

[4] Tadayonnejad, R., Yang, S., Kumar, A.,Ajilore, O. Multimodal brain connectivity analysis in unmedicated late-life depression. *PLoS One* https://doi.org:10.1371/journal.pone.0096033 (2014).

[5] Meng, Y., Li, H., Wang, J., Xu, Y.,Wang, B. Cognitive behavioral therapy for patients with mild to moderate depression: Treatment effects and neural mechanisms. *J Psychiatr Res* https://doi.org:10.1016/j.jpsychires.2021.02.001 (2021).

[6] Kerestes, R., Harrison, B. J., Dandash, O., Stephanou, K., Whittle, S., Pujol, J. *et al.* Specific functional connectivity alterations of the dorsal striatum in young people with depression. *Neuroimage Clin* https://doi.org:10.1016/j.nicl.2014.12.017 (2015).

[7] Gong, L., He, C., Yin, Y., Wang, H., Ye, Q., Bai, F. *et al.* Mediating Role of the Reward Network in the Relationship between the Dopamine Multilocus Genetic Profile and Depression. *Front Mol Neurosci* https://doi.org:10.3389/fnmol.2017.00292 (2017).

[8] Ang, Y. S., Kaiser, R., Deckersbach, T., Almeida, J., Phillips, M. L., Chase, H. W. *et al.* Pretreatment Reward Sensitivity and Frontostriatal Resting-State Functional Connectivity Are Associated With Response to Bupropion After Sertraline Nonresponse. *Biol Psychiatry* https://doi.org:10.1016/j.biopsych.2020.04.009 (2020).

[9] Gong, L., Yin, Y., He, C., Ye, Q., Bai, F., Yuan, Y. *et al.* Disrupted reward circuits is associated with cognitive deficits and depression severity in major depressive disorder. *J Psychiatr Res* https://doi.org:10.1016/j.jpsychires.2016.09.016 (2017).

[10] Wang, L., An, J., Gao, H. M., Zhang, P., Chen, C., Li, K. *et al.* Duloxetine effects on striatal resting-state functional connectivity in patients with major depressive disorder. *Hum Brain Mapp* https://doi.org:10.1002/hbm.24601 (2019).

[11] Liu, R., Wang, Y., Chen, X., Zhang, Z., Xiao, L.,Zhou, Y. Anhedonia correlates with functional connectivity of the nucleus accumbens subregions in patients with major depressive disorder. *Neuroimage Clin* https://doi.org:10.1016/j.nicl.2021.102599 (2021).

[12] Gabbay, V., Ely, B. A., Li, Q., Bangaru, S. D., Panzer, A. M., Alonso, C. M. *et al.* Striatum-based circuitry of adolescent depression and anhedonia. *J Am Acad Child Adolesc Psychiatry* https://doi.org:10.1016/j.jaac.2013.04.003 (2013).

[13] Chen, G., Chen, P., Gong, J., Jia, Y., Zhong, S., Chen, F. *et al.* Shared and specific patterns of dynamic functional connectivity variability of striato-cortical circuitry in unmedicated bipolar and major depressive disorders. *Psychol Med* https://doi.org:10.1017/S0033291720002378 (2020).

[14] Hou, Z., Gong, L., Zhi, M., Yin, Y., Zhang, Y., Xie, C. *et al.* Distinctive pretreatment features of bilateral nucleus accumbens networks predict early response to antidepressants in major depressive disorder. *Brain Imaging Behav* https://doi.org:10.1007/s11682-017-9773-0 (2018).
